# Fabrication of Ethosomes Containing Tocopherol Acetate to Enhance Transdermal Permeation: In Vitro and Ex Vivo Characterizations

**DOI:** 10.3390/gels8060335

**Published:** 2022-05-30

**Authors:** Naheed Akhtar, Naveed Akhtar, Farid Menaa, Walaa Alharbi, Fatima Saad Salem Alaryani, Ali Musfer Alqahtani, Faizan Ahmad

**Affiliations:** 1 Department of Pharmacy, Islamia University of Bahawalpur, Bahawalpur 63100, Pakistan; naheedakhtar@upr.edu.pk; 2 Department of Nanomedicine, California Innovations Corporation, San Diego, CA 92037, USA; 3 Department of Chemistry, Science and Arts College, King Abdulaziz University, Rabigh Campus, Jeddah 21911, Saudi Arabia; wnhalharbe@kau.edu.sa; 4 Department of Biology, Faculty of Sciences, University of Jeddah, Jeddah 21959, Saudi Arabia; fsalaryani@uj.edu.sa; 5 Department of Pharmacology, College of Pharmacy, King Khalid University, Guraiger, Abha 62529, Saudi Arabia; amsfr@kku.edu.sa; 6 Department of Pharmacy, Bahawalpur Medical and Dental College (BMDC), Bahawalpur 63100, Pakistan; faizahmadBMDC@gmail.com

**Keywords:** tocopherol acetate, ethosomes, gel formulation, permeation studies, drug delivery, cosmetics

## Abstract

Background: Tocopherol acetate (TA) is known as a skin moisturizing and photoprotective agent. One major drawback with tocopherol and its derivatives remains its limited stability. Aim: To develop highly stable TA-containing ethosomal gel (TAEG) as an advanced dosage form. Methods: A cold method technique was used to produce the ethosomes. An in vitro evaluation of viscosity, conductivity, and pH stability was carried out for three months. An in vitro physical characterization of the nanoparticles (NPs) that included particle size (PS), zeta potential (ZP), transmission electron microscopy (TEM), and Fourier-transform infrared (FTIR) spectroscopy analysis was then performed. Organoleptic evaluation, thermostability at 8 °C, 25 °C, 40 °C and 40 °C ± 75% RH, pH, conductivity, viscosity, and spreadability measurements were also performed in vitro for three months. An ex vivo permeation study was performed in phosphate-buffered solution (1× PBS; pH 5.5 or pH 7.4) at 37 ± 0.2 °C by using rat abdominal skin and the Franz diffusion cell method. The data of three independent experiments were expressed as mean ± SD. A two-way ANOVA was applied to compare data on TAEG versus TA control gel (TACG). Results: PS of the ethosomes was in the range of 144–289 nm. A total of nine formulations were developed. Optimized TAEG formulation (TA-5) was selected based on the highest entrapment efficiency (EE) of 99.71%, while the stability, the PS, and the uniformity-based polydispersity index (PDI) were also among the best. TA-5 exhibited smooth spherical ethosomal NPs with PS of 200.6 nm, ZP value of −18.6 V, and PDI of 0.465. Stability data obtained for TA-5 in terms of rheology, conductivity, and pH presented no significant change (*p* > 0.05) during the entire study duration. Rheological studies indicated that TA-5 followed a non-Newtonian behavior of shear thinning system. The ex vivo drug permeation was 44.55 ± 0.01% in TA-5 and the drug retention in skin was 51.20%, which was significantly higher than TACG as observed after 24 h permeation study (*p* < 0.05). Conclusions: The newly developed TAEG formulation appears promising to enhance the effectivity of TA and its topical application.

## 1. Introduction

The human body is covered by many layers of skin, which is the largest organ and naturally protects the body from continuous exposure to intense hazardous environmental factors (e.g., heat, cold, temperature, light, microbes, and dirt particles) [1]. Skin also provides a route for topical drug delivery, for both local and systemic effects [2]. Importantly, the stratum corneum is the outermost layer of the skin, which helps to prevent and regulate the water content and other salts in body fluids [2,3].

**Figure 1:**
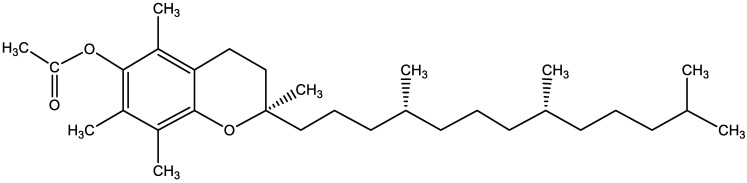
Chemical structure of α-tocopherol acetate (C_31_H_52_O_3_).

Tocopherol has a crucial role in the skin physiological structure and functions [4]. It is an important component of the biological membrane, acting as a lipophilic antioxidant skin [4]. The derivatives of vitamin E are known as tocols, which belong to the family of tocotrienols and tocopherols [4]. The primary structure of tocols is a 6-hydroxy-2-methyl-phytylchroman. In nature, there are eight known tocols divided into four tocopherols and four tocotrienols further classified as alpha (α), beta (β), gamma (γ), and delta (δ) isomers. Esterified form is more stable against oxidation then free or non-ester form. Vitamin E derivatives commercially available are tocopherol acetate (TA), tocopherol succinate (TOS) and tocopherol polyethylene glycol-1000 succinate [5]. Tocopherols are liposoluble [6]. TA is the most biologically active form of vitamin E [7]. The molecular formula of TA is C_31_H_52_O_3_, and its molecular weight is 472.7 g/mol (Figure 1). Its melting point is 26.5–27.5 °C. It is a slightly yellow or off-white crystalline substance which is odorless, sensitive to light and heat. TA is an extremely weak basic substance with a pKa value of 4.9. TA is insoluble in water but forms a clear solution in acetone and methanol. TA and TOS are the most used derivatives compared with other tocopherol derivatives [8].

In terms of applications, tocopherol and its ester forms are present in many cosmetics products for skin moisturizing and as antioxidant agents. For instance, alpha-TA represents the main lipophilic antioxidant in many sunscreens and commercial dermatological products (e.g., skin conditioning agents, humectants, anti-aging solutions) to prevent transepidermal water (TEWL/TWL) loss and aging [9]. Nevertheless, it is worth mentioning that different vitamin E compounds show various degrees of photoprotection when topically used at the concentration of 5% [10]. Usually, 1–5% concentrations of TA is used in many topical products to protect skin and treat skin conditions such as atopic dermatitis [9,11]. Increases in TA concentrations from 5% to 10% is not correlated with increases in skin permeability and retention on skin [9]. TA helps to prevent skin from dangerous effects of UV radiation [12]. Since TA is depleted with continuous exposure to UV radiation [13], skin health maintenance is required by percutaneous administration of the lipophilic antioxidant TA.

Interestingly, it has been reported that vitamin E and its derivatives are not only used in cosmetics (e.g., as photoprotective agents) but also in combination with other nanomedicines to improve the solubility and therapeutic effects of poorly water-soluble drugs [14]. Therefore, various vitamin E derivatives are used as adjuvants for the treatment of cancer to improve the solubility of antineoplastic agents [5]. Several research studies evidenced the antitumor activity exerted by orally (*per os*) administrated vitamin E and derivatives [15]. So far, a wide range of drug delivery systems have been developed due to the valuable biological and physicochemical properties of tocopherol derivatives [16,17]. Indeed, tocopherols were used in nanoformulations, including liposomes, NPs, niosomes, and nanoemulsions, for oral and topical applications. The active development and use of these nanodrug delivery systems (NDDS) have solved many issues related to skin permeation, biodistribution, bioavailability, absorption, stability, metabolism, and solubility [17]. Permeation and metabolism of TA is highly depending on the type of formulation and thus on the drug delivery system [17]. This subsequently increased their concentration at the desired site of action with a minimal drug dosage, thereby eliminating unwanted side effects linked to drug overdose, whereas the therapeutic index is enhanced [18].

Ethosomes was developed for the first time in 1997 [19,20]. Ethosomes are artificially prepared as self-assembled spherical, stable, and soft lipid vesicles [21]. Ethosomes are basically composed of 20–45% alcohol (ethanol or isopropyl alcohol) relatively with high amount of phospholipids, and purified water. The ethosomes are then produced from the combination of ethanol, propylene glycol, phospholipids, and water [20]. Structurally, ethosomes are like liposomes, which is a conventional drug delivery system consisting of layered lipids, but ethosomes contain higher concentration of ethanol than ethosomes [22]. Thanks to their enhanced physicochemical characteristics (e.g., stability, EE), ethosomes are suitable for the efficient transport, controlled and tissue-targeting drug delivery of lipophilic, hydrophilic, and amphiphilic drugs into deep layers of skin [23]. Additionally, this vesicular system is much smaller than liposomes (from tens of nanometers to a few microns) and depicted with unilamellar or multilamellar structure when visualized by TEM [24,25]. Research studies reported that ethosomes containing a stable formulation with good EE increase permeation and percutaneous absorption [26]. Ethosomes were found to be a more efficient delivery carrier, with high encapsulation capacities (98.5% ± 1.4%) and PS (200.6 ± 1.6 nm) [27,28]. In vitro transdermal permeation experimental studies showed that the permeation of tocopherol through abdominal skin of albino rat skin is significantly higher when ethosomes was used as carrier of delivery system. The solid vesicular drug nanocarriers range from 1 to 1000 nm [29]. Ethosomes increased the deposition of TA into skin to reduce hyperpigmentation and dehydration of skin efficiently [30]. Fabrication of developed ethosomes into semisolid dosage form increases stability, enhance penetration and increased retention into epidermis with improved beneficial effects [30]. Previous studies found that ethosomal vesicles were stable for 90 days at room temperature (RT), and ethosomal vesicles presented unchanged EE value, sedimentation, or phase separation [20]. Hence, we sought that incorporation of TA into ethosomes could have many advantages over other conventional drug delivery systems.

The aim of this work is to develop TAEG as novel transdermal NDDS for potentializing the effects of TA. In vitro and ex vivo characterizations have been further carried out before any in vivo investigations.

## 2. Materials and Methods

### 2.1. Reagents

Tocopherol acetate and propylene glycol was purchased from Sigma-Aldrich Chemie GmbH (Taufkirchen, Germany); Cholesterol was obtained from AppliChem GmbH (Darmstadt, Germany); Soy phosphatidylcholine 90 G were obtained from Lipoid GmbH (Ludwigshafen, Germany). Carbopol-940 and Ethanol by Merck KGaA (Gernsheim, Germany). All the chemicals and materials used for ethosomes vesical development were of analytical standard. Double distilled water (ddH_2_O) was used throughout the study.

### 2.2. Instruments

The instruments used in this study include a sonicator/ultrasonic homogenizer (EP100H, Elma Ultrasonic, Ruiselede, Belgium), centrifuge machine (Hettich EBA 20, Sigma-Aldrich Chemie GmbH, Taufkirchen, Germany), portable conductivity/pH meter (ProfiLine^™^ Cond 197i, VWR, Visalia, CA, USA), electrical balance (BJ-210, Precisa, Poissy, France), heating magnetic stirrer (VELP scientifica Srl, Usmate, Italy), Zetasizer (Nano Series ZEN3600, Malvern Instruments Ltd., Worcestershire, UK), digital rheometer (DV-III Ultra, Brookfield Engineering Laboratories, Inc., Stoughton, MA, USA), FT-IR spectrometer (Bruker Tensor 27, SpectraLab Scientific Inc., Markham, ON, Canada), hot incubator (Sanyo MIR-162, Gemini Lab B.V., Apeldoorn, The Netherlands), UV-Vis spectrophotometer (Irmeco U2020, Irmeco GmbH & Co. KG, Luejensee, Germany),Transmission Electron Microscope Jem 1010 (Jeol USA, Inc., Peabody, MA, USA), and Franz diffusion cell (PermeGear, Inc. # 4G-01-00-15-12, Hellertown, PA, USA).

### 2.3. Preparation of Ethosomal NPs

Ethosomes were produced using modified cold method technique [28]. All required chemical and material were first selected and weighed accurately. Lipid-soluble chemicals were dissolved in a measured amount of ethanol, and the TA drug was dissolved into the previous mixture with continuous stirring in a closed container at RT. Propylene glycol was used as penetration enhancer and added into the above non aqueous phase, then this mixture was heated on water bath at 30 °C. The pre-heated aqueous phase, in separate vessels at 30 °C, was added to the above non aqueous phase as small droplets with syringe under stirring constantly for 5 min. The mixture was cooled at RT to allow the formation of ethosomal vesicles. The size of the developed vesicles was reduced by stirring for 30 min at 750 rpm and by sonication for 30 min using an ultra sonicator [31]. Desired size vesicles were obtained by the extrusion method. The uniformly sized developed ethosomal vesicle suspension was stored in a refrigerator (4 °C) for further analysis and experimental purposes [24,32].

### 2.4. Preparation of Ethosomal Gels

1% *w*/*w* Carbopol 940 was used as a gelling agent for the preparation of TAEG. Accurately weighed amount of carbopol was sprinkled in a measured quantity of distilled water (dH_2_O) to soak for overnight [31]. Triethanolamine was added drop by drop with homogenous stirring with homogenizer until a clear transparent homogenized gel having pH between 6–6.5 was formed. Optimized TA containing ethosomal suspension was added to the gel slowly with continuously mixing by using homogenizer to obtained uniform gel formulation contained ethosomal vesicles [28,30]. A total of 9 formulations were developed, but in a pre-screening study, TA-5 formulation was selected as the optimized formulation based on increased EE, ZP, PS, and PDI.

### 2.5. Physicochemical Characterizations of Ethosomal Dispersions

#### 2.5.1. Zeta Potential (ZP) and Particle Size (PS)

Suspended NPs were characterized for their average PS and electrostatic potential of charge, which are important features for stability studies [33,34]. The PS and ZP of the dH_2_O diluted TA-5 formulation were assessed by dynamic light scattering (DLS) using a Zeta Sizer Nano ZS90, Malvern, UK at 25 °C. The experiments were triplicated independently to minimize the risk of errors.

#### 2.5.2. Drug Entrapment Efficiency (% EE)

The drug entrapment efficiency calculated how much amount of the drug was entrapped into developed spherical vesicles. The % EE was calculated by simple indirect analysis technique [23,33,34]. First, 1 mL ethosomal suspension was centrifuged for 30 min to obtain a clear supernatant solution. The collected supernatant was then diluted with 0.2 M phosphate-buffered solution pH 7.4, which was freshly prepared. This procedure was performed thrice. The supernatant (test sample) was eventually analyzed at 295 nm by UV spectrophotometry. By using the formula given below, the percent (%) EE was determined:

$$\%\;\mathrm{EE}=\frac{\mathrm{Total}\;\mathrm{amount}\;\mathrm{of}\;\mathrm{drug}-\mathrm{Amount}\;\mathrm{of}\;\mathrm{free}\;\mathrm{drug}}{\mathrm{Total}\;\mathrm{amount}\;\mathrm{of}\;\mathrm{drug}}\times 100$$

#### 2.5.3. Transmission Electron Microscopy (TEM)

Optimized ethosomal TA-5 dispersion was analyzed for its morphology by TEM [34]. The analysis was performed by negative stain procedure [13]. Briefly, the test sample solution was diluted 1:10 with dH_2_O and sonicated for 5 min. Then, a drop of the diluted test sample solution was put on carbon-coated copper grid and air dried at RT, keeping the test sample on grid for 1 min. For staining, the test sample, 2% (*w*/*v*) phosphotungstic acid was dropped on copper grid, and the extra stain was removed by using tissue paper before TEM analysis was performed at 60 KV.

#### 2.5.4. Organoleptic, PH, Conductivity, and Spreadability Assessments

TA ethosomal gel (TAEG) and TA control gel (TACG) were assessed for organoleptic features (i.e., appearance of gel, color, odor, and feel after use of these gels) as well as for their relative thermostability at 8 °C, 25 °C, 40 °C and 40 °C ± 75% RH, temperatures and spreadability for three months (90 days) [30]. Spreadability evaluation was performed by using glass slide method [1]. Briefly, 0.5 g gel was placed on 1 cm circled marked glass slide by putting another glass slide over the first slide. Weight of 500 g was placed on this upper slide for 5 min and a scale was used to measured increased gel diameter [13,35].

#### 2.5.5. Rheological Analysis

TAEG and TACG were analyzed by using a programmable rheometer, with spindle number CP41, and Rheocalc version 2.5.6. Viscosity studies are important to determine the stability of a semisolid system [1]. To analyze the thermostability of viscosity of both TAEG and TACG, they were placed at different temperatures for three months. Each sample was analyzed for shear stress, viscosity, and shear rate. Results were obtained as average ± standard deviation (SD) from three independent experiments. A sample of each gel (0.5 ± 0.01 g) was placed in a sample holding cup and analyzed at 20–100 rpm [1].

### 2.6. Ex Vivo Permeation Studies

This study was performed using Franz cell method and albino rat abdominal skin, as the closest human-like natural skin membrane, on which TAEG and TACG were applied [36]. First, the rat skin was obtained and washed with normal saline solution. Fatty tissues were separated carefully, and the skin was then cut into circular form according to the size of the Franz cell circumference. Skin was mounted on the cell in such a way that stratum corneum face the donor chamber (compartment). Donor compartment, already filled with 1× PBS of pH 5.5 (human skin pH), was maintained at 37 ± 0.2 °C using a water bath. Continuous stirring was performed to keep the temperature constant in Franz cell attached with the water bath. About 0.5 g of each gel was applied on the mounted skin. Then, 3 mL of the sample was withdrawn at per scheduled time duration from the donor compartment and, to maintain the volume, the same volume of prewarmed 1× PBS of pH 5.5 was added to the donor compartment, so that the skin remains in contact with the solution in the donor compartment. The study was performed for 24 h, and the obtained samples were analyzed at 295 nm by UV spectrophotometry [36]. The same procedure was performed at pH 7.4 (human systemic/blood pH) to determine the effects of pH on permeation.

### 2.7. Statistical Analysis

The obtained results were evaluated statistically by using IBM SPSS Statistics version 20 software (IBM, Armonk, NY, USA) for paired sample t-test analysis and two-way ANOVA. The IBM SPSS statistic version 20 was used for the statistical analysis of the data during the course study. Paired sample *t*-test was applied to observe a potential difference between two formulations. The results were considered statistically significant if the *p*-values were less than 5% (*p* < 0.05). The data were expressed as mean values of ±standard error of mean (SEM).

## 3. Results and Discussion

### 3.1. Fabrication and Optimization of TAEG Formulations

Ethosomes have been included as a vesicular-based NDDS. Ethosomes are hydroalcoholic preparations enabling encapsulation of both hydrophilic and hydrophobic drugs [27]. Ethosomes can entrap high amount of drugs, including insoluble or poorly water-soluble compounds, due to their high EE, which is an asset for controlled drug delivery [37].

TAEG and TACG formulations were fabricated by a simple cold method technique [31]. This method is simple, effective, and easy to perform. It consists of a simple mixing and a careful dropping of the aqueous phase into the nonaqueous phase, before reducing the PS by sonication and extrusion [38].

To obtain the highest stability and EE [39], ethosomal vesicles were produced by varying the concentration of lipid and ethanol while keeping other constituents’ concentration constant (Table 1). In a pre-screening study, the test formulation (TA-5) has produced optimal characteristics, including EE (Table 1), and was then selected for further in vitro and ex vivo investigations. EE of the optimized TA-5 formulation was 99.71% due to its high lipophilicity [40]. Then, 30% ethanol was used to formulate TA-5. These findings are in line with previous research on tocopherols (98–100% EE) [39,40]. Such high EE can be explained by [41,42] (i) the increased number of C-H bonds formed between lipophilic drugs and hydrophobic tail structures, (ii) the membrane formed from ethanol or phospholipid, which have good potential characteristics affinity for both lipophilic and hydrophilic drugs to solubilize and encapsulate, and (iii) the membrane hydrophobic nature, which also permits a sustained drug release. Like other vesicles, such ethosomes act as drug depot, and are considered as stable vesicles increasing skin permeation [43].

### 3.2. Physicochemical Characterizations of TA-Loaded Ethosomes

#### 3.2.1. TEM Analysis

The results obtained by TEM analysis of ethosomal formulation depicted round shaped ethosomes (Figure 1). TEM is a technique that uses an electron beam to image a NP sample, providing much higher resolution than is possible with light-based imaging techniques. Indeed, TEM is the preferred method to directly measure nanoparticle size, grain size, crystallographic structure, size distribution, chemical composition, and morphology [44].

**Figure 2:**
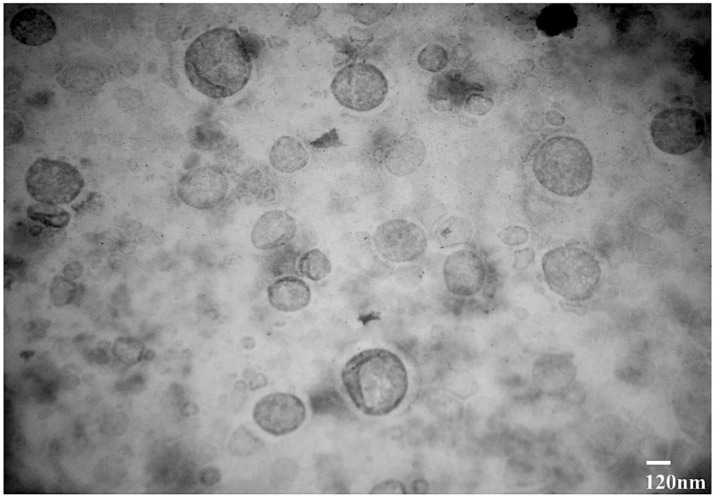
TEM micrograph of the optimized TAEG formulation (TA-5). Scale bar is indicated.

TEM micrographs of TA-5 confirm the spherical morphology of formed vesicles and nano size of uniform ethosomal particles (Figure 2). As showed in Table 1 and Table 2, spherical shaped and nonporous smooth surface multilamelar ethosomes were produced by combination of 30% ethanol and 2.5% phospholipid with enhanced and high entrapped drug concentration. These data are in line with other studies reporting the fabrication of ethosomal gel particles [21,45].

#### 3.2.2. PS Analysis

Interestingly, we found that the decreasing amount of phospholipid cause vesicles size decreases (Table 2), and subsequently, smaller vesicles entrapped less amount of drug. PS has a great influence on drug disposition, diffusion, release factors and deposition on skin [46]. There are many factors (e.g., chemical structure, nature and amount of drug and lipid used) and experimental techniques which impact the PS and the amount of drug loaded in NPs [7,47]. The PS and EE have a direct relationship in terms of ethosomes formation, and so, by controlling these factors it is possible to improve the stability and bioavailability of many drugs. The amount of hydrophobic TA drug entrapment is due to the electrostatic attraction of oppositely charged ethosomes and TA drug molecule. The EE of larger size ethosomes is higher than small size particulate vesicles [48]. Additionally, the combination of phospholipid and ethanol enhanced the ethosomal EE compared to the use of ethanol alone or phospholipid alone [49]. Eventually, the high EE of TA is further due to its high lipophilicity, because all the added drug amount in the inner lipophilic core is kept [50]. Herein, we found that by increasing the lipid concentration, the size of vesicles increases with increased EE, an observation which agrees with previous studies [13].

#### 3.2.3. PDI Analysis

The PDI of a formulation is a key parameter for the characterization of NPs uniformity in the disperse system. PDI ranging between 0.1 and 0.7 indicates homogeneous dispersion and narrow size distribution [13,51]. PDI value greater than 0.7 indicates broad size distribution [13,51]. The PDI of the prepared TAEG formulations was found in the range of 0.214–0.475, which therefore indicates high uniformity PSD (Table 2).

#### 3.2.4. ZP Analysis

ZP indicates the electrostatic stability of colloidal disperse systems [34,52]. Indeed, ZP represents the potential difference between the electronic charged ions on particles, which is a very important parameter to prevent NPs aggregation [31,34,36]. This charge may be positive or negative. ZP ranged from −16.8 (mV) to −28.4 (mV) at RT (Table 2), so all the nine TA ethosomal formulations were physically stable at this temperature condition (*p* > 0.05).

**Table 2:** Particle size (PS), polydispersibility index (PDI), and zeta potential (ZP) of the TAEG formulations (N = 9).

#	Formulation Code	Ethanol (%)	Phospholipid (%)	PS (nm)	PDI	ZP (mV)
1	TA-1	25	5	235	0.475	−18.9
2	TA-2	25	2.5	159	0.377	−21.7
3	TA-3	25	0.5	144.4	0.312	−19.2
4	TA-4	30	5	289	0.389	−20.6
**5**	**TA-5**	**30**	**2.5**	**200.6**	**0.465**	**−18.6**
6	TA-6	30	0.5	156	0.277	−16.8
7	TA-7	40	5	231.2	0.361	−28.4
8	TA-8	40	2.5	207.6	0.348	−20.3
9	TA-9	40	0.5	153.7	0.214	−17.9

#### 3.2.5. FT-IR Analysis

**Figure 3:**
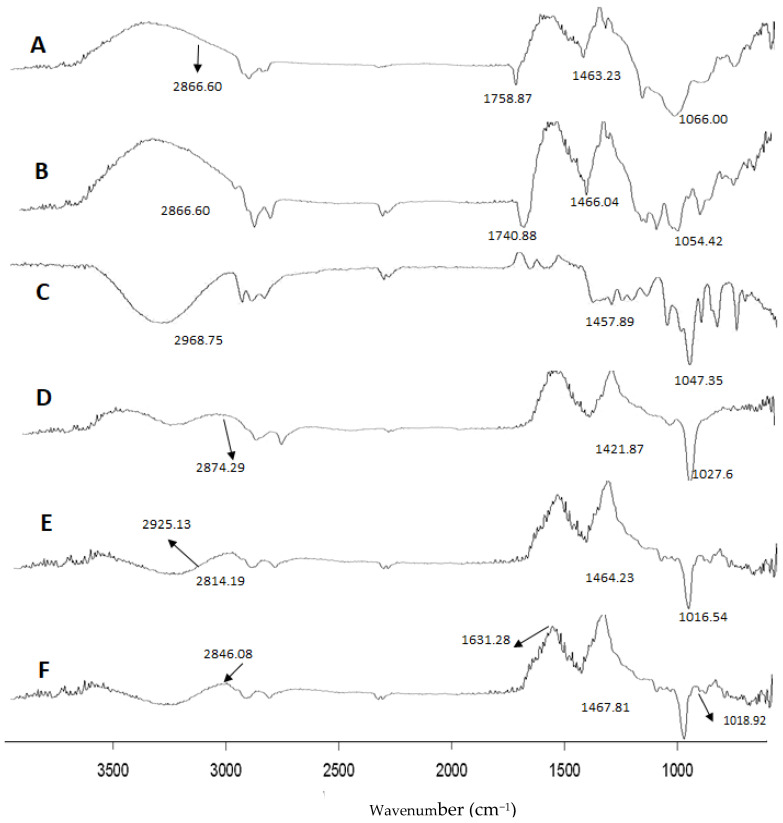
FTIR spectra of TA (**A**), PC (**B**), PEG (**C**), Ethanol (**D**), TACG (**E**), and TAEG (TA-5) (**F**).

FTIR study were performed on the free TA drug, phosphatidylcholine (PC, aka lecithin), propylene glycol (PEG), Ethanol, TACG, and TAEG (TA-5) in the range of 400–4000 cm^−1^, as shown in Figure 3.

The peaks at 1366.67 cm^−1^, 1377.14 cm^−1^, 1368.12 cm^−1^ are attributed to CH_3_ stretch in TAEG, PEG and PC, respectively. The peaks at 1463.23 cm^−1^, 1457.89 cm^−1^, 1466.04 cm^−1^ are assigned to CH_2_ bend in TAEG, PEG and PC, respectively.

The peaks at 2866.60 cm^−1^, 2925.13 cm^−1^, 2874.29 cm^−1^ are attributed to C–H stretches of TA drug, PEG, and PC, respectively.

The peaks at 1758.87 cm^−1^, 1740.88 cm^−1^ would indicate C=O stretch in TAEG and PC, respectively. The peaks at 1066.00 cm^−1^, 1064.42 cm^−1^ would represent C-O stretch in TAEG and PC, respectively.

In PC, the peaks at 1630 cm^−1^ and 1064.42 cm^−1^ are attributed to C–N and P=O stretches, respectively.

In TAEG, the peak at 685.63 cm^−1^ is assigned to C=O stretch in plan bending. The peaks at 1018.92 cm^−1^ and 1019.32 cm^−1^ in TAEG is assigned to C–O stretch in the carboxylic group. The peak at 1467.81 cm^−1^ in TAEG is assigned to CH_3_ bend, which is slightly shifted from 1366.67 cm^−1^. The peak at 1479.8 cm^−1^ in TAEG belongs to CH_2_ bend. The peaks at 1631.28 cm^−1^ and 1642.33 cm^−1^ in TAEG are attributed to C=C stretch of benzene or phenyl. The sharp peak at 1754.86 cm^−1^ in TAEG is attributed to C=O bend of the ester group. The wide peaks at 2925.04 cm^−1^ and 2846.08 cm^−1^ in TAEG belong to C-H bends.

The sharp peak at 1632.26 cm^−1^ in TACG is attributed to C=O bend of the ester group. The peaks at 1633.18 cm^−1^ and 1652.23 cm^−1^ in TACG are attributed to C=C stretch of benzene or phenyl.

The FTIR spectrum of TAEG formulation demonstrated that there was no new peak formation that occurs in the active drug and formulation ingredients. Thus, we can conclude that there was no chemical interaction between the active drug and ethosomal formulation components.

#### 3.2.6. Thermostability, and Organoleptic Analysis

The optimized TAEG (TA-5) and TACG (control) formulations were evaluated for organoleptic characteristics, thermostability, pH, conductivity, and viscosity (Table 3). TA-5 and TACG were placed at various temperatures (i.e., 8 °C, 25 °C, 40 °C, 40 °C ± 75% RH) for 90 days.

TA-5 and TACG were similarly stable initially at 8 °C and 25 °C during the full study period (*p* > 0.05). Furthermore, we observed that, at 40 °C and 40 °C ± 75% RH, both TA-5 and TACG started to become light yellow from the second month (*p* > 0.05), whereas TACG started to become slightly more yellow than TAEG at the third month. Nevertheless, there were not enough significant differences (*p* > 0.05) between TA-5 and TACG in terms of thermostability in the last month at 40 °C and 40 °C ± 75% RH.

Additionally, no odor and liquefaction were produced in both TA-5 and TACG during the entire study period (*p* > 0.05).

Smoothness was unchanged in the first 2 months but, at the third month, TA-5 and TACG gels became slightly thin (*p* > 0.05) at 40 °C and 40 °C ± 75% RH.

Taken together, data showed no significant difference (*p* > 0.05) when comparing the TA-5 to the control, demonstrating that the optimized ethosomal formulation is stable and that the loading of TA into the ethosomal gel had no significant stability effects.

#### 3.2.7. pH, Conductivity, and Viscosity Analyses

**Figure 4:**
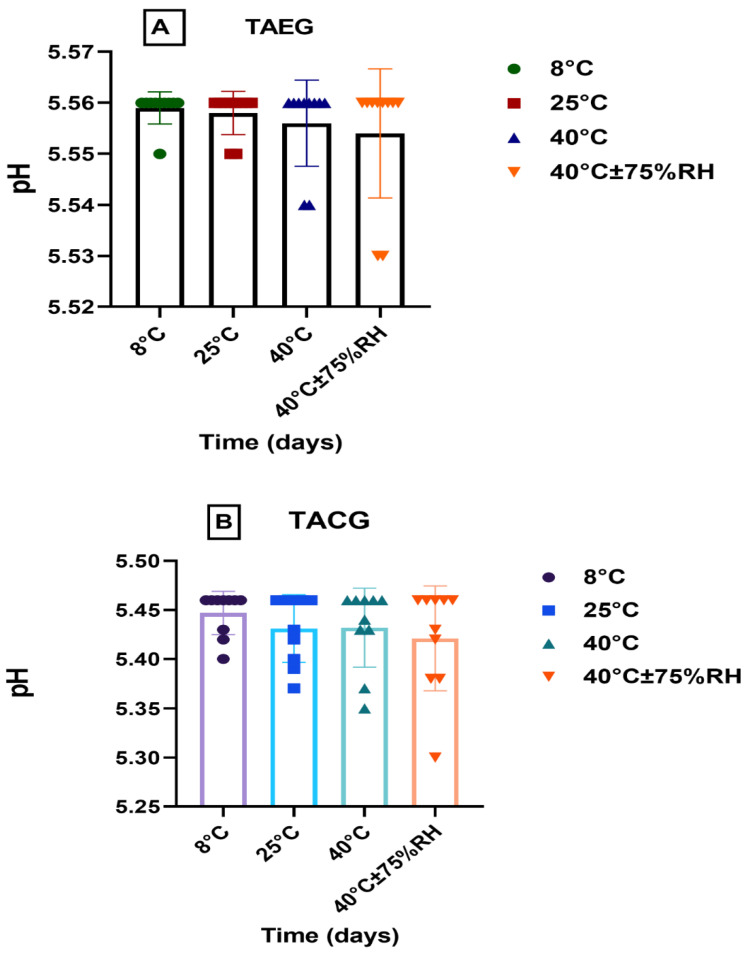
pH of (**A**), TAEG, and (**B**), TACG, kept at different temperatures for the study period of three months. Data are expressed as mean values ± SEM. Insignificant results were produced at 8 °C and 25 °C, 40 °C, and 40 °C ± 75% RH (*p* > 0.05).

**Figure 5:**
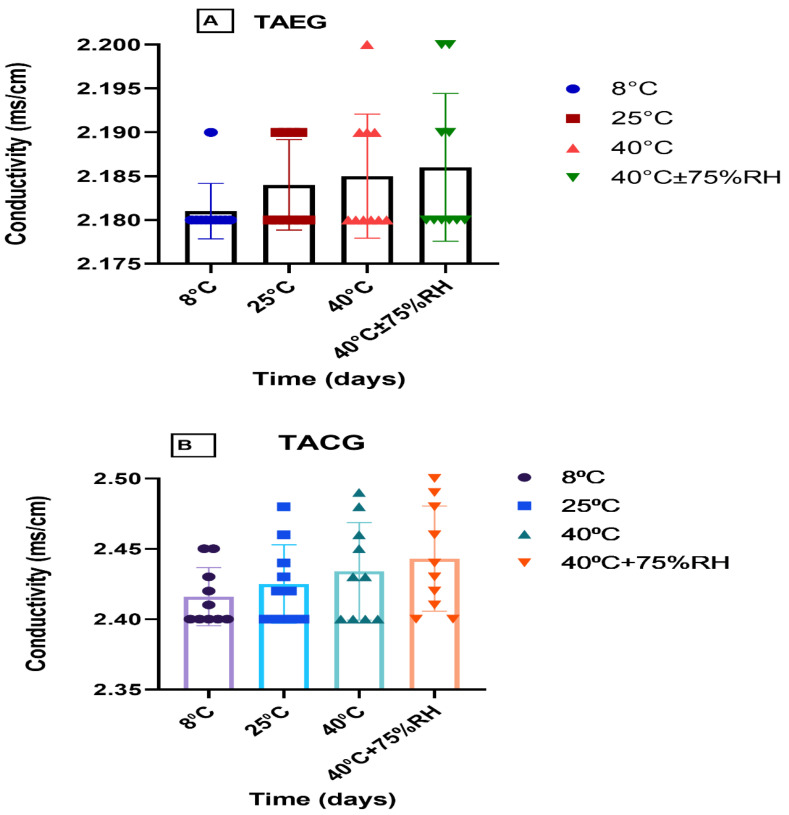
Conductivity of (**A**), TAEG, and (**B**), TACG, kept at different temperatures for the study period of three months. Data are expressed as mean values ± SEM. Insignificant results were produced at 8 °C and 25 °C, 40 °C, and 40 °C ± 75% RH (*p* > 0.05).

**Figure 6:**
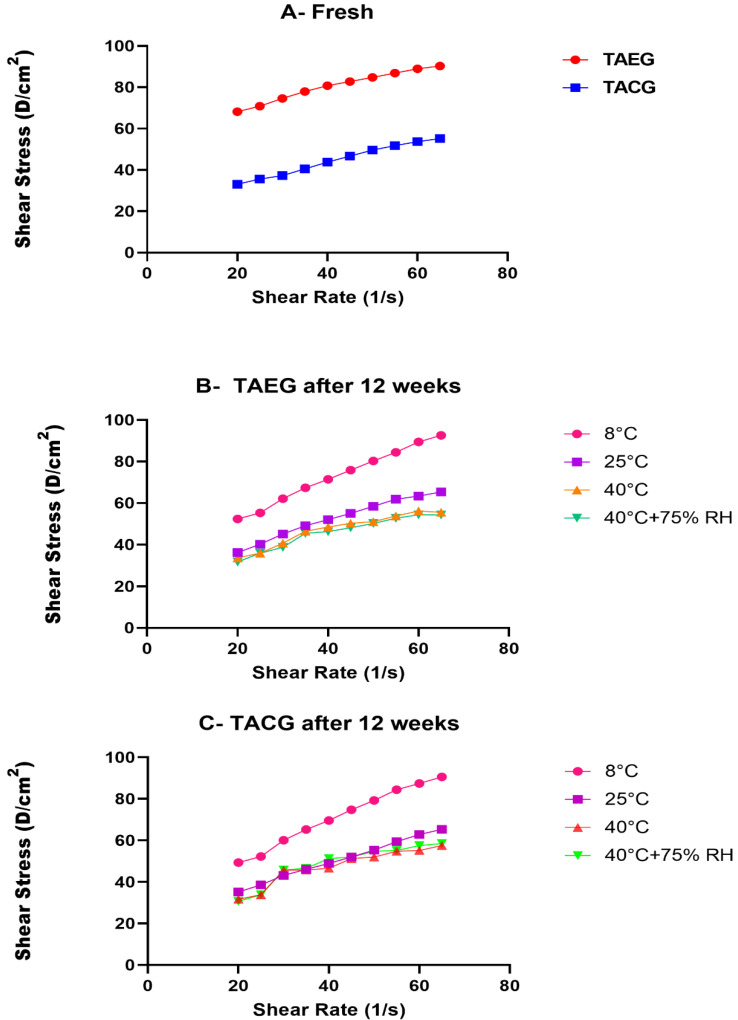
Rheological analysis of (**A**), Optimized TAEG (TA-5) and TACG freshly prepared at room temperature (25 °C), (**B)**, Optimized TAEG (TA-5) after 12 weeks, (**C)**, TACG, after 12 weeks. The formulations were kept at different temperatures 8 °C, 25 °C, 40 °C and 40 °C ± 75% RH and at share rate of 1/s. Data are expressed as mean values ± SEM. Shear thinning effects were produced, but insignificant results are obtained once the study period is completed (*p* > 0.05).

The pH, rheological (viscosity), and conductivity analyses were carried out for the optimized TAEG (TA-5) and TACG, which were kept at various temperatures (i.e., 8 °C, 25 °C, 40 °C, 40 °C ± 75% RH) for 12 weeks (90 days/3 months), as represented in Figure 4, Figure 5 and Figure 6, respectively.

Although the conductivity of the freshly prepared TA-5 was significantly higher than TACG (*p* > 0.05%) at RT (Figure 6A), we could observe that, after 12 weeks, their pH (Figure 4A,B, respectively) and viscosity (Figure 6B,C, respectively) similarly decreased slightly (*p* > 0.05) while the conductivity similarly increased (Figure 5A,B, respectively).

When the paired sample *t*-test was applied, significant (*p* ≤ 0.05) changes in conductivity was observed between TA-5 and TACG (Figure 5A,B, respectively). When the two-way ANOVA was applied, insignificant differences (*p* > 0.05) were noticed in the conductivity of TA-5 with respect to time and temperature (Figure 5A); however, significant (*p* ≤ 0.05) changes in conductivity were observed for TACG with respect to time and temperature (Figure 5B).

### 3.3. Ex Vivo Permeation Analysis

Various important information was obtained from ex vivo study analyses of the optimized TAEG (TA-5) and TACG formulations at both pH 5.5 (Human skin-like) and pH 7.4 (human blood-like) (Table 4). Important parameters such as flux, coefficient of permeability of TA-5 and TACG were evaluated; percent drug retained on skin was also studied. Targeting efficiency was determined and analysis of enhancement ratio was performed.

**Figure 7:**
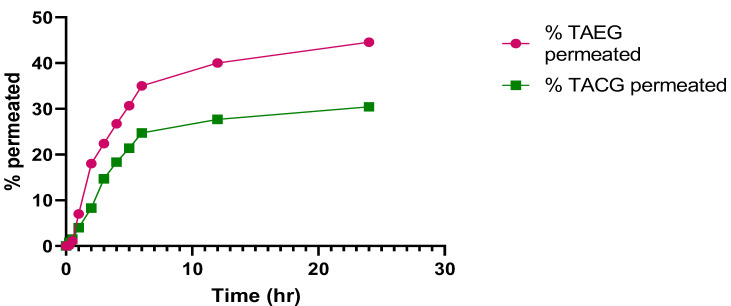
Time-dependent percentage permeation of optimized TAEG (TA-5) and TACG at physiological human skin pH 5.5 and at physiological human temperature (37 °C). Significative differences were observed (*p* < 0.05).

**Figure 8:**
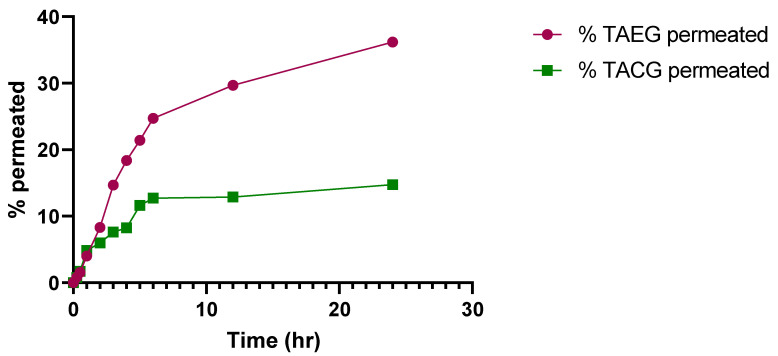
Time-dependent percentage permeation of optimized TAEG (TA-5) and TACG at physiological human skin pH 7.4 and at physiological human temperature (37 °C). Significative differences were observed (*p* < 0.05).

The permeation of TA at pH 5.5 from TA-5 was 44.55 ± 0.01%, which was significantly higher (*p* < 0.05) than permeation of TACG (30.44 ± 0.01%), as represented in Figure 7. The permeation (%) of TA at pH 7.4 from TAEG was 36.18 ± 0.02%, which was significantly higher (*p* < 0.05) compared to that of permeation of TACG (14.76 ± 0.02%), as represented in Figure 8. The data obtained in these two conditions of human body-like pH demonstrated a better permeation of TA-5 at pH 5.5 compared to that of pH 7.4 (*p* < 0.05), strongly suggesting an increased retention of the drug in the skin rather than in the circulation. This could be explained by a lower drug systemic penetration. Therefore, such formulation should be indicated for topical application rather than for intravenous (IV) administration. The TA-5 flux value was greater compared to that of TACG.

Indeed, at pH 5.5 and 37 °C, the enhancement ratio (ER) indicated that TA permeation from TA-5 was increased by 1.17 compared to TACG, whereas ER was increased only by 0.44 at pH 7.4 and at the same physiological human body-like temperature (*p* < 0.05), as summarized in Table 4. Furthermore, at pH 5.5 and 37 °C, TA-5 was more efficient in targeting (ratio 2.15) compared to TACG, whereas TE was increased only by 1.30 at the same temperature but at pH 7.4 (*p* < 0.05), as shown in Table 4.

According to the kinetic modeling, TA-5 showed increased R^2^ values of zero-order model compared to the first-order model. Therefore, it can be assumed that TA will be released at a constant rate from TA-5 independent of the amount remaining in TA-5. Overall, a best fit of release data was obtained from Higuchi model (R^2^ = 0.9091–0.9944) confirming a Fickian diffusion-controlled release mechanism. Conformingly, the drug release exponent of Korsmeyer–Peppas model (n) was less than 0.45, with appropriate regression coefficient (R^2^ = 0.9867), allowing us to definitively conclude to the Fickian diffusion-based drug release [13,53]. Therefore, through ethosomal gel, TA followed the Fick’s law of diffusion.

The hydrophilic and adhesive nature of the gelling agent used in this study, i.e., Carbopol-940, as well as appropriate physical properties and the effective permeation of TA from TA-5 through Fickian diffusion collectively strongly suggest that TA-derived ethosomal gel can effectively be utilized for the topical delivery for the local action [54].

Similar results were seen in other studies where the ethosomal gel has presented higher permeability than the control gel [48,55]. The reason for increased permeation from ethosomes is more likely due to nanosized vesicles and properties of phospholipid used to prepare ethosomes [56,57]. Other permeation studies reported that the percentage retention of TA in the skin is greater from ethosomal gel compared to simple drug solution [58]. Additionally, it is worth mentioning that ethosomes are non-irritant to skin in comparison to niosomes [13,31].

## 4. Conclusions

Herein, a stable TAEG formulation was successfully developed. The TA ethosomal gel (TAEG) carrier TA-5 had the best EE as well as adequate PS and uniformity. Additionally, the formulation displayed a good skin retention capacity with increased enhancement ratio and high targeting efficiency. The formulation elicited prolonged action for sustained drug delivery. This study suggests that ethosomes could be modified to obtain the desired sustained delivery of TA effects simply by using different concentrations of ethanol and phospholipid amount. Taken together, the novel ethosomal system was found to be a valuable NDDS for topical TA delivery and might be used for therapeutic and cosmetic purposes.

## Figures and Tables

**Table 1:** TAEG formulations.

Code	Drug (%)	Propylene Glycol (%)	Phospholipid (%)	Ethanol (%)	Water (%)	E.E (%)
TA-1	0.25	10	5	25	q.s	91.80
TA-2	0.25	10	2.5	25	q.s	92.03
TA-3	0.25	10	0.5	25	q.s	96.37
TA-4	0.25	10	5	30	q.s	99.2
**TA-5**	**0.25**	**10**	**2.5**	**30**	**q.s**	**99.71**
TA-6	0.25	10	0.5	30	q.s	97.89
TA-7	0.25	10	5	40	q.s	93.04
TA-8	0.25	10	2.5	40	q.s	95.78
TA-9	0.25	10	0.5	40	q.s	97.73

q.s: Quantum satis.

**Table 3:** Thermostability of TAEG (TA-5, E) and TACG (C) at 8 °C, 25 °C, 40 °C, and 40 °C ± 75% RH for a study period of three months (90 days).

Observed Parameters	Temp.	Fresh		After 48 h		After 72 h		After 7 days		After 21 days		After 30 days		After 60 days		After 90 days	
		C	E	C	E	C	E	C	E	C	E	C	E	C	E	C	E
**Color**	**8 °C**	W	W	W	W	W	W	W	W	W	W	W	W	W	W	W	W
	**25 °C**	W	W	W	W	W	W	W	W	W	W	W	W	W	W	W	W
	**40 °C**	W	W	W	W	W	W	W	W	W	W	OW	W	LY	LY	Y	LY
	**40 °C** **± 75% RH**	W	W	W	W	W	W	W	W	W	W	OW	W	LY	LY	Y	LY
**Odor**	**8 °C**	-	-	-	-	-	-	-	-	-	-	-	-	-	-	-	-
	**25 °C**	-	-	-	-	-	-	-	-	-	-	-	-	-	-	-	-
	**40 °C**	-	-	-	-	-	-	-	-	-	-	-	-	-	-	-	-
	**40 °C** **± 75% RH**	-	-	-	-	-	-	-	-	-	-	-	-	-	-	-	-
**Look**	**8 °C**	T	M	T	M	T	M	T	M	T	M	T	M	T	M	T	M
	**25 °C**	T	M	T	M	T	M	T	M	T	M	T	M	T	M	T	M
	**40 °C**	T	M	T	M	T	M	T	M	T	M	T	M	T	M	T	M
	**40 °C** **± 75% RH**	T	M	T	M	T	M	T	M	T	M	T	M	T	M	T	M
**Liquefaction**	**8 °C**	-	-	-	-	-	-	-	-	-	-	-	-	-	-	-	-
	**25 °C**	-	-	-	-	-	-	-	-	-	-	-	-	-	-	-	-
	**40 °C**	-	-	-	-	-	-	-	-	-	-	-	-	-	-	-	-
	**40 °C** **± 75% RH**	-	-	-	-	-	-	-	-	-	-	-	-	-	-	-	-

T = Transparent, M = Milky, W = White, OW = Off White, LY = Light yellow, Y = yellow, Negative (-) = No change, C = Control, E = Ethosomes.

**Table 4:** Permeability coefficient, enhancement ratio (ER), targeting efficiency (TE), drug retention, and flux values of optimized TAEG (TA-5) compared to TACG.

Formulations	pH 5.5			ER of TAEG	TE of TAEG
	Flux (µg/cm².h)	Papp (cm/h)	% Drug Permeated		
TAEG	23.75	0.0095	44.55 ± 0.01%	1.173	2.15
TACG	20.25	0.0081	30.44 ± 0.01%		
	**pH 7.4**				
TAEG	3.43	0.0014	36.18 ± 0.01%	0.441	1.30
TACG	7.72	0.0031	14.76 ± 0.01%		

## Data Availability

The data presented in this study are available on request from the corresponding author.
